# A call-to-action for sustainability in dialysis in Brazil

**DOI:** 10.1590/2175-8239-JBN-2019-0014

**Published:** 2019-06-27

**Authors:** José A. Moura-Neto, Katherine Barraclough, John W. M. Agar

**Affiliations:** 1 Grupo CSB, Salvador, Bahia, Brasil.; 2 Department of Nephrology, Royal Melbourne Hospital, Parkville, Australia.; 3 University of Melbourne, Parkville, Australia.; 4 Renal Unit, University Hospital Geelong, Victoria, Australia.

**Keywords:** Hemodialysis Units, Hospital, Renal Dialysis, Environment and Public Health, Conservation of Natural Resources, Peritoneal Dialysis, Nephrology, Unidades Hospitalares de Hemodiálise, Diálise Renal, Meio Ambiente e Saúde Pública, Conservação dos Recursos Naturais, Diálise Peritoneal, Nefrologia

## Abstract

Human-induced climate change has been an increasing concern in recent years. Nephrology, especially in the dialysis setting, has significant negative environmental impact worldwide, as it uses large amounts of water and energy and generates thousands of tons of waste. While our activities make us responsible agents, there are also several opportunities to change the game, both individually and as a society. This call-to-action intends to raise awareness about environmentally sustainable practices in dialysis and encourages this important discussion in Brazil.

## RESUMO

A mudança climática induzida pela atividade humana tem sido foco de preocupações crescentes nos últimos anos. A nefrologia, particularmente a diálise, produz significativos impactos ambientais em todo o mundo em virtude da grande utilização de água e energia e da geração de milhares de toneladas de resíduos. Embora nossas atividades nos tornem agentes responsáveis, há várias oportunidades para mudar esse cenário, tanto individualmente como em sociedade. O presente artigo pretende ampliar a conscientização sobre práticas ambientalmente sustentáveis em diálise e estimular essa importante discussão no Brasil.

## INTRODUCTION

The planet has experienced major changes in the past decades as a result of human activities. Humans are now present on every continent and have had direct impact on more than 80% of the planet’s surface^1^; living, interacting globally with the environment, bringing developments to humankind, but also doing some harm.

Relevant to the medical community, climate change has been identified as the greatest human health threat of this century. It is increasingly leading to alterations in the patterns of diseases, including renal disease.^2^ This brings some threats, but also an opportunity while there is still time to critically evaluate our actions and look at current healthcare practices in order to mitigate the environmental impact.

## DISCUSSION

The healthcare sector, paradoxically, has a significant and negative environmental impact. Its contribution to greenhouse gas emissions, for example, is estimated to be between 5 and 10%. In the United States of America (USA), the healthcare sector produces more than 8% of the national carbon dioxide (CO_2_) emissions^3^, while in the United Kingdom, the National Health Service (NHS) is responsible for approximately 5%.^4^ The healthcare sector is responsible for an estimated 35,772 kt of CO^2^ emissions in Australia^5^, which corresponds to 7% of all national emissions.

The nephrology specialty, and especially the dialysis sector, is among one of the most environmentally harmful within the healthcare system. More than 3,000,000 patients worldwide are estimated to be on maintenance dialysis programs. This number is projected to more than double in the next years and to exceed 7,000,000 patients by 2030. Despite the impressiveness of this number, it would be at least two times bigger if equal and adequate access to healthcare were globally available and dialysis were universally provided. Unfortunately, what is known as the “renal replacement therapy gap” caused between 2.3 and 7.1 million premature deaths because of lack of access to renal replacement therapy.^6^^-^ 8

In dialysis, water usage is one of the most wasteful aspects. Patients undergoing conventional hemodialysis (HD), in a 4-hour thrice-weekly regimen, are estimated to use around 500 liters of water per treatment. This estimate is based on the assumption that each HD treatment, with an average dialysate flow rate of 500 mL/min, requires around 125 L of water per patient, which represents only one-third of the total water used **-** most reverse osmosis systems reject approximately two-thirds of the total water. In addition to this value, we must add the volume of sterilization, pre-HD priming, and post-HD rinsing, which may reach 500 L per treatment. In addition, it is important to note that this value may vary depending not only on the frequency and length of sessions, but also on the reverse osmosis system and HD machine type.^9^^,^ 10

Remarkably, this represents an annual water usage of around 78,000 L per patient, compared with the average annual domestic water consumption per capita of approximately 42,500 L in Brazil 11, 54,500 L in Australia^12^, and 55,000 L in France^13^. We can estimate an HD water consumption of more than 200 billion liters globally based on the total number of patients receiving HD treatment.^14^^,^ 15

Peritoneal dialysis (PD), the renal replacement therapy utilized by around 10% of patients worldwide^16^ and 8% in Brazil^17^, probably wastes less water, requiring between 6 to 12 liters per PD dialysate per day.^9^^,^ 14 However, there is no study that estimates the amount of water necessary to produce this sterile fluid, and the total water usage in PD is probably much greater than 12 liters per day.

In addition to water waste, hemodialysis is estimated to consume approximately 2 billion kWh of power annually^15^ and 1 million tons of disposable waste **-** which includes dialyzers, lines, needles, and other single-use equipment.^18^ Taken together, these numbers turn dialysis into one of the greatest waste-producing and resource-demanding practices in the healthcare sector.

The question that we should ask ourselves is: what can be done with this scenario? How can we act to reduce our environmental impact as care providers? The answers come partially from eco-dialysis or green dialysis initiatives, which have been gaining force worldwide. The main idea of this initiative is to discuss ways to minimize the environmental impact of dialysis practices. Although it is recent, some nations, such as Australia, the Netherlands, and the United Kingdom, are far beyond others and have already made interesting moves towards more sustainable dialysis practices. In the United Kingdom, for example, the National Health Service Sustainable Healthcare program established the Green Nephrology initiative in 2009, which has successfully changed practices and attitudes throughout the region.^19^ However, even in these countries, there is still an opportunity to further adopt sustainable practices in the dialysis setting. In Australia, a recent survey of 71 public dialysis facilities in the state of Victoria showed that only 10% of the units used renewable sources of energy, solar in these cases, and 25% recycled reverse osmosis reject water for use elsewhere, such as to water gardens or flush toilets.^20^ Although these countries are likely much ‘greener’ than other countries for which we do not have this baseline data yet, there is still much to be improved.

During the American Society of Nephrology 2018 Conference (San Diego, USA), a global meeting on “Green Nephrology” was held for the first time. The meeting, which was supported by the International Society of Nephrology (ISN), was attended by nephrologists and representatives from various medical societies around the world and discussed several ways to engage the renal community worldwide. The meeting led to the following initiatives: the proposal of Green Nephrology as a topic for a scientific session at the 2020 World Congress of Nephrology and the elaboration of an ISN position statement by this group, headed by Dr Katherine Barraclough. All these recent events are very positive and possibly an indication that sustainability may be a future trend in renal replacement therapy globally, as is already the case in other areas.

Basically, there are three major targets for an initial approach in the green dialysis setting. We shall first focus on power, water usage, and dialysis waste management.^9^ There are already several publications that address ways to reduce water waste^10^^,^ 21 ^-^ 23 and encourage the use of solar energy.^10^^,^ 24 Power-off practices, the use of natural light, and lighting choices (incandescent, fluorescent or light-emitting diode - LED) are several options to reduce the overall use of energy.^14^ Other possibilities of sustainable practices for dialysis facilities exist, such as reducing the use of paper and printed files, encouraging staff to share automobiles (car-pooling) or use bikes, and using telemedicine or teleconferencing for patient consultations and meetings to avoid carbon emissions from travel. Most of these practices are economically viable and even profitable for dialysis facilities, which makes convincing the board of directors and investors to support a sustainable approach much easier. In a scenario in which the costs of renal replacement therapy around the world are an increasing concern and our capability as a healthcare system to universally provide this therapy is at risk^25^^,^ 26, this are indeed excellent news. After all, green dialysis is a win-win initiative; advantageous for the environment, society, and dialysis facilities.

Although beneficial to the environment, as it reduces the risks and cost of infectious-risk dialysis waste disposal, it is important to note that the reuse of equipment raises concerns related to the quality of the dialysis performed and the clinical staff’s chemical exposure. In fact, reuse of dialyzers and lines is discouraged and illegal in some countries, such as Australia, Japan, New Zealand, France, Italy, and Germany. However, it is still practiced in the USA and Brazil - which has been a matter of increasing debate.^14^^,^ 27 ^,^ 28

In Brazil, there are 758 dialysis facilities and 126,583 chronic patients receiving maintenance dialysis.^29^ This number increases each year and represents an important impact to the environment. If we consider that 92.1% of the patients are under HD^17^ and a total of 116,583 chronic HD patients in Brazil performing 156 HD sessions annually each, then we can conservatively estimate a total of approximately 9 billion L/year of water used for HD in Brazil alone (500 L/patient/treatment x 116.583 patients x 156 treatments/year). Most of this amount, approximately two-thirds, is constituted of reusable and potable water - usually discarded in the drain.^14^ Additionally, if we consider that each patient on thrice-weekly hemodialysis maintenance annually generates 323 kg of waste^30^, we can estimate that hemodialysis generates a total of 37,656 tons of waste in Brazil (116,583 patients x 323 kg/patient/year).

Furthermore, with a better healthcare system, this number will probably increase because more patients will have access to renal replacement therapy, which is a current trend in developing countries. Therefore, it is important to discuss ways to reduce the environmental impact of dialysis practice. However, there is still a paucity of discussion about this subject in Brazil.^31^

## CONCLUSION

As healthcare providers, one of our ultimate goals must be “*primum non nocere*”, which means, “first, to do no harm”.^32^ Dialysis is an established treatment that, while saving millions of lives, paradoxically causes great negative impact to the environment that may affect the health and wellbeing of billions of people. Sooner or later, climate change will force us to redesign our practices and reduce the harm we cause through governmental laws and regulations. We must anticipate this move and be proactive; under these circumstances, it is better to lead than to be led.^33^

Green or eco-dialysis is still little discussed in Brazil, and nephrologists and stakeholders are uncertain about what this really means. The first step for a change in current practice is to spread the word of the necessity of sustainability in dialysis throughout the country to create a collective consciousness. Green dialysis is not only a concept or a theoretical discussion. Quite the opposite, there are many tangible and practical opportunities available - as outlined above - to effectively reduce our environmental footprint. We hope that this “Call-to-Action” may cause an epiphany in relation to the harm we have been causing as nephrologists and be a turning point for developing an environmentally positive attitude towards a sustainable future.
